# The complete mitochondrial genome of the hybrid grouper *Epinephelus akaara*♀ × *Epinephelus lanceolatus*♂

**DOI:** 10.1080/23802359.2018.1473727

**Published:** 2018-05-21

**Authors:** Yin Guo, Weiping Li, Yali Liu, Peisi Li, Runping Wei, Yun Liu, Yong Zhang, Ling Xiao

**Affiliations:** State Key Laboratory of Biocontrol, Institute of Aquatic Economic Animals and Guangdong Provincial Key Laboratory for Aquatic Economic Animals, School of Life Sciences, Sun Yat-Sen University, Guangzhou, China

**Keywords:** Mitochondrial genome, *Epinephelus akaara*♀ × *Epinephelus lanceolatus*♂

## Abstract

In this study, the complete mitochondrial genome of *Epinephelus akaara* × *Epinephelus lanceolatus* has been presented. The mitochondrial genome is 16,795 bp long and consists of 13 protein-coding genes, 2 rRNA, 22 tRNA, and a D-loop region. The phylogenetic analysis by neighbour-joining (MJ) method showed that the hybrid grouper has the closer relationship to *E. akaara*.

*Epinephelus akaara* and *E. lanceolatus* are both belong to Serranidae in the ordo perciformes. Both of them have high economic value and are widely distributed in South China, Korea, southern China, and Southeast Asian countries. The hybrid grouper F1 is inclined to take *E*. *akaara* as female parent and *E*. *lanceolatus* as male parent. As the hybrid generation has a faster growth and a better disease resistance, and there is little information of F1 genetic characteristics, the next-generation sequencing technique was used to analysis the complete mitochondrial genome. The specimen of F1 generation was obtained from the Marine Fisheries Development Center of Guangdong Province, China. The total genomic DNA was extracted from the fin of the fresh fish using the salting-out procedure (Howe et al. 1997).

The complete mitochondrial genome of *E. akaara* × *E. lanceolatus* is 16,795 bp in length (Genebank, KY132101), consisting of 13 protein-coding genes, 2 rRNA, 22 tRNA, and a D-loop region. Most of the genes are encoded on the heavy strand, with only the DADH dehydrogenase subunit 6 and eight tRNA genes are encoded on the light strand. The composition nucleotide of light strand is 27.28% for A, 28.68% for T, 27.84% for G, and 16.2% for C, and the heavy strand is 28.68% for A, 27.28% for T, 16.20 for G, and 27.84 for C.

We perform multiple sequence alignment and construct a neighbour-joining (MJ) phylogenetic tree (Figure 1) (Tamura et al. 2013). As shown, the hybrid grouper was closer with the *E. akaara*.

**Figure 1.**
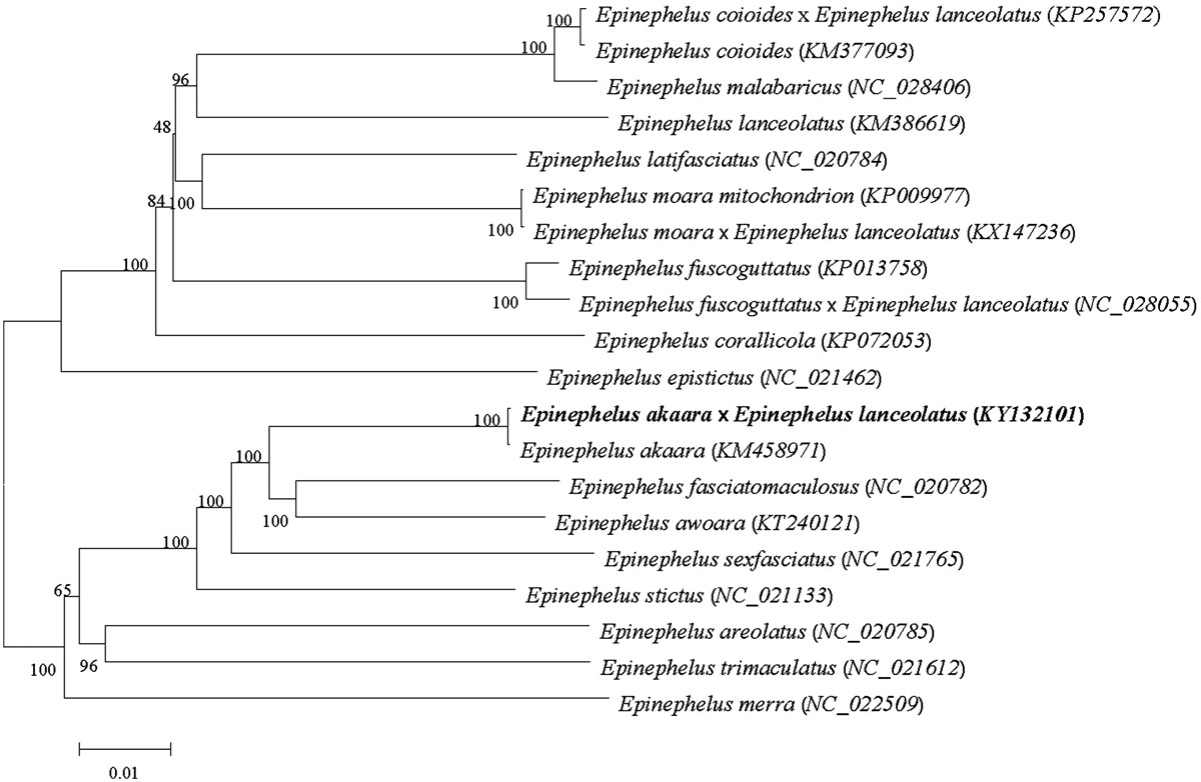
The NJ phylogenetic tree of Perciformes species. Numbers on each node are bootstrap values of 100 replicates.
